# Management of Adverse Events and Supportive Therapy in Relapsed/Refractory Multiple Myeloma

**DOI:** 10.3390/cancers13194978

**Published:** 2021-10-04

**Authors:** Samantha Pozzi, Alessia Bari, Martin Pecherstorfer, Sonia Vallet

**Affiliations:** 1 Department of Medical and Surgical Sciences for Children & Adults, University Hospital of Modena and Reggio Emilia, Largo del Pozzo 71, 41124 Modena, Italy; samantha.pozzi@unimore.it (S.P.); alessia.bari@unimore.it (A.B.); 2 Department of Internal Medicine 2, University Hospital Krems, Mitterweg 10, 3500 Krems, Austria; martin.pecherstorfer@krems.lknoe.at; 3 Karl Landsteiner University of Health Sciences, Dr. Karl-Dorrek-Straße 30, 3500 Krems, Austria; 4 Molecular Oncology and Hematology Unit, Karl Landsteiner University of Health Sciences, Dr. Karl-Dorrek-Straße 30, 3500 Krems, Austria

**Keywords:** relapsed/refractory multiple myeloma, supportive care, treatment toxicity

## Abstract

**Simple Summary:**

Multiple myeloma (MM) patients with relapsing and/or refractory (RR) disease are exposed for a prolonged time to multiple drugs, which increase the risk of toxicity. In addition to tumor response, preserving the quality of life represents an important goal for this patient population. Therefore, supportive therapy plays a pivotal role in their treatment by limiting disease- and drug-related complications. The aim of this review is to outline current standards and future strategies to prevent and treat renal insufficiency, anemia, bone disease, and infection, including COVID-19, in RRMM patients. In addition, the incidence and treatment of side effects of novel anti-MM agents will be discussed.

**Abstract:**

Relapsed/refractory (RR) multiple myeloma (MM) patients are a fragile population because of prolonged drug exposure and advanced age. Preserving a good quality of life is of high priority for these patients and the treatment of disease- and treatment-related complications plays a key role in their management. By preventing and limiting MM-induced complications, supportive care improves patients’ outcome. Erythropoietin-stimulating agents and bisphosphonates are well-established supportive strategies, yet novel agents are under investigation, such as anabolic bone agents and activin receptor-like kinase (ALK) inhibitors. The recent dramatic changes in the treatment landscape of MM pose an additional challenge for the routine care of RRMM patients. Multidrug combinations in first and later lines increase the risk for long-lasting toxicities, including adverse cardiovascular and neurological events. Moreover, recently approved first-in-class drugs have unique side-effect profiles, such as ocular toxicity of belantamab mafodotin or gastrointestinal toxicity of selinexor. This review discusses current standards in supportive treatment of RRMM patients, including recommendations in light of the recent SARS-CoV-19 pandemic, and critically looks at the incidence and management of side effects of standard as well as next generation anti-MM agents.

## 1. Introduction

With an estimated incidence rate of 2.1 per persons worldwide, multiple myeloma (MM) is one of the most common hematological malignancies [1]. Clinical manifestations of MM range from a dysfunctional immune system, impaired hematopoiesis, and bone fragility to organ failure, including the kidney, heart, and nervous system. Complications of MM are not only a source of morbidity, but they are the leading cause of death [2]. Indeed, advances in supportive therapies along with the integration of novel strategies in the treatment landscape of MM have significantly contributed to the improved survival observed in the recent decade [3,4,5].

Despite therapeutic progress, up to 20% of newly diagnosed patients present with primary refractory disease and almost all of the responding patients eventually relapse [6,7]. Due to the prolonged exposure to therapies with overlapping toxicities, relapsed/refractory (RR) MM patients are a vulnerable population, often characterized by a limited bone marrow reserve and decreased renal function and peripheral neuropathy. In addition to disease control, an adequate quality of life is a priority of second-line treatment and beyond. Indeed, the side effects of next-generation anti-MM agents may jeopardize treatment adherence and negatively influence survival [8].

This review outlines current recommendations and future strategies for the supportive care of RRMM patients with renal failure, anemia, and bone disease, and discusses the challenges of the rapidly evolving treatment landscape in MM.

## 2. Renal Disease

Renal impairment (RI), defined as creatinine clearance (CrCl) below 40 mL/min, is observed in more than one third of RRMM patients and is associated with a higher incidence of adverse events and a 20% increase in the risk of disease progression or death [9,10,11]. The most common form of RI in MM is cast nephropathy due to the precipitation of excessive free light chain. In addition, hypercalcemia, hyperuricemia, and nephrotoxic agents may exacerbate RI. The treatment of acute MM-related RI consists in the rapid initiation of anti-tumor therapy to reduce the light chain burden and of adequate supportive care to eliminate contributing factors. A high-dose of dexamethasone and volume repletion under constant monitoring of fluid balance are recommended. In case of hypercalcemia, the concomitant administration of denosumab may improve renal function. In addition, nephrotoxic agents should be avoided, including aminoglycoside antibiotics, non-steroidal anti-inflammatory drugs, furosemide, and contrast agents [12]. Despite discordant results from randomized trials, free light chain removal through high-cutoff hemodialysis should be considered in MM patients requiring dialysis because of the acute kidney injury due to light chain cast nephropathy [13,14,15].

The presence of RI limits anti-MM treatment options and precludes the inclusion in most of the clinical trials. Retrospective analysis and small prospective studies have evaluated safety and efficacy of anti-MM treatments, including proteasome inhibitors (PI), immunomodulatory agents (IMiDs), chemotherapy, and, more recently, monoclonal antibodies, in RRMM patients with renal failure [9,16,17,18]. Nephrotoxic medications, such as lenalidomide and zoledronic acid, require specific dosing adjustments, as indicated in the FDA and EMA drug approval specifications. On the other hand, some agents, especially PI, are preferred in the treatment of RRMM patients with RI for their renal protective effect and rapid hematologic response (Table 1). Recent data confirms the efficacy of PI-based regimens for the renal rescue of RRMM patients with RI [9,12]. A post hoc exploratory subgroup analysis of the ENDEAVOR study showed a rate of renal response with bortezomib and carfilzomib (defined as improvement of CrCl ≥ 60 mL/min in patients with baseline CrCl < 50 mL/min) of approximately 15% [9]. Similarly, in a recent real-world study including more than 1500 patients with RRMM and RI, renal complete response was 26.6% in patients treated with carfilzomib/dexamethasone and 22.2% with bortezomib/dexamethasone [19]. Interestingly, PI-free combination therapies may also induce renal recovery, as recently shown in the ICARIA-MM study. A prespecified subgroup analysis demonstrated long-lasting renal rescue in more than 30% of patients treated with isatuximab, pomalidomide, and dexamethasone [20]. The efficacy and safety of daratumumab in combination with bortezomib and dexamethasone is currently evaluated in RRMM patients with severe renal failure (GMMG-Dante, NCT).

## 3. Anemia

Anemia is a frequent finding in RRMM, with hemoglobin values below 11 g/dL in more than 40% of the patients. The pathogenesis of anemia is multifactorial: direct toxic effect of MM cells, chronic inflammatory state, and renal disease may reduce bone marrow reserves, as well as drug-induced myelosuppression [23,24]. Indeed, the incidence of anemia ranges from 15% in patients treated with daratumumab or carfilzomib to 60% with selinexor [25,26,27].

Current treatment options for MM-associated anemia include red blood cell (RBC) transfusions and erythropoiesis-stimulating agents (ESAs). RBC are rapidly but only transiently effective, therefore indicated for the acute management of symptomatic patients or asymptomatic high-risk patients [28]. ESAs, such as epoetin or darbepoetin alpha, provide a sustained increase in hemoglobin, thus reducing the need for RBC transfusion [24]. However, ESAs increase the risk of thromboembolic events in MM patients, especially in combination with IMiDs and dexamethasone [29,30,31]. Therefore, treatment with ESAs should follow international guideline recommendations and initiated only after a careful assessment of risk and benefit [32].

Preclinical studies and early clinical trials are investigating alternative strategies against MM-associated anemia by targeting the activin signaling pathway or the chemokine CCL3. Activin receptor ligand traps, such as sotatercept and luspatercept, enhance erythroid differentiation by downregulating the SMAD2/3 signaling in hematopoietic progenitor cells [33]. Sotatercept, in combination with melphalan, prednisolone, and thalidomide, demonstrated a bone anabolic and erythropoietic effect in MM patients [34]. Luspatercept has been recently approved for the treatment of ESA-resistant anemia in patients with myelodysplastic syndrome [35]. Interestingly, the inhibition of the activin receptor-like kinase (ALK)-2 improves anemia in mouse models of iron-refractory/iron-deficiency anemia by repressing hepcidin, a critical mediator of anemia of chronic disease [36,37]. A novel ALK-2 inhibitor, INCB000928, is currently assessed in a phase 1/2 trial in patients with myelodysplastic syndrome or MM who are transfusion-dependent or have symptomatic anemia (NCT04582539). Finally, a recent study suggests that MM-derived CCL3 suppresses the expression of GATA1, a master regulator of erythropoiesis, by binding to CCR1 on hematopoietic stem and progenitor cells. The inhibition of the CCL3/CCR1 pathway may therefore represent an additional promising strategy to overcome anemia in MM [23].

## 4. Bone Disease

Osteolytic bone disease in MM may lead to severe skeletal-related events (SREs), including hypercalcemia, pain, bone fractures, as well as the need for surgery or radiotherapy. Bone lesions are detected in 80% of patients with newly diagnosed MM and SREs occur in 40%. Despite exposure to bone-protecting agents, more than 20% of MM patients at first relapse experience SREs, thus also highlighting the significant economic and clinical burden of bone disease in the relapsed/refractory setting [38].

Therapeutic approaches for painful bone lesions or fractures consist of local and systemic strategies. In particular, kyphoplasty is recommended for symptomatic vertebral compression fracture, surgery for long-bone pathological fractures or vertebral column instability, and radiotherapy should be considered for uncontrolled pain or spinal cord compression [39]. To treat and prevent SREs, two classes of antiresorptive agents are available: bisphosphonates (BPs) and the RANKL inhibitor denosumab. Zoledronic acid (ZA) is the preferred BP because of the survival advantage compared to clodronate in newly diagnosed MM and is recommended for all patients regardless of the presence of myeloma-related bone disease on imaging [32,39,40,41,42]. In particular, the most recent guidelines of the Bone Working Group of the International Myeloma Working Group (IMWG) support resuming ZA at time of asymptomatic biochemical relapse [39]. As shown in the AZAPACHE study, the treatment of patients with biochemical MM relapse with ZA did not show antitumor effects, yet significantly prevented occurrence of SREs [43]. Importantly, osteoprotective treatment should not be interrupted in refractory MM patients. 

Although monthly ZA is the preferred bone-targeted strategy in MM, nephrotoxicity limits its use in patients with RI [44]. A valid alternative in case of renal failure is denosumab, which showed in newly diagnosed MM patients no-inferiority for time to first SRE compared to ZA, with significantly less renal adverse events [45]. In contrast to ZA, Denosumab is currently recommended only for MM patients with evidence of bone disease, in particular if renal failure is present [39]. Of note, since its approval, the use of denosumab in MM increased dramatically, regardless of renal function or BP-intolerance, mainly because of the advantages of subcutaneous administration, raising questions on costs and long-term toxicity [46]. Similarly to ZA, denosumab has no anti-MM effects in the relapsed setting but effectively suppresses markers of bone resorption [47].

The treatment recommendations for hypercalcemia of malignancy include ZA, as first-agent, and denosumab, for BP-refractory patients. However, only BPs are currently licensed in Europe after denosumab application withdrawal from AMGEN in January 2017 [48,49]. In addition, hydration, high-dose steroids, and calcitonin may also be useful in the presence of hypercalcemia. 

Side effects of anticatabolic agents include hypocalcemia and osteonecrosis of the jaw (ONJ). Since incidence of hypocalcemia is 12% in ZA-treated and 17% in denosumab-treated patients, calcium and vitamin D supplements are required during treatment. ONJ is a rare, yet serious complication occurring in 2–6% of the patients [50]. To reduce the risk for ONJ in MM patients, in addition to regular dental status controls, a maximal treatment duration with osteoprotective agents of two years is recommended. In addition, after one year of treatment with standard-dose ZA, responders could be offered a 3-monthly dosing. In a large phase III randomized trial, no difference in SREs was observed in MM patients treated with ZA every 4 or 12 weeks [51]. Similarly, the Z-MARK study confirmed the safety of a less intense ZA schedule in patients with low of urinary N-telopeptide of type 1 collagen (uNTX), a marker of bone turnover [52,53]. Retrospective analyses suggest similar results with denosumab, yet the lack of prospective studies restricts the use of a less-frequent dosing of denosumab only within clinical trials [54]. Since rechallenge with ZA or denosumab in the setting of relapsed MM may lead to ONJ recurrence, the risk/benefit balance of osteoprotective treatment should be carefully discussed [55].

Despite continuous or previous exposure to osteoprotective agents, RRMM are still at high risk of skeletal complications and current research efforts are directed to the development of bone anabolic agents to implement skeletal stability in MM. Inhibitors of activin A, DKK1 and sclerostin stimulate differentiation of osteoblasts, which are responsible for bone formation [56,57,58,59]. In a phase 1B study, the inclusion of the DKK1 inhibitor BHQ880 to standard therapy and ZA in RRMM increased bone mineral density in the lumbar spine [60]. Similarly, the activin A antagonist sotatecerpt, in combination with melphalan, prednisolone, and thalidomide, improves bone mineral density and bone formation in RRMM [34]. Further studies are warranted to confirm the benefit of these novel agents in MM.

## 5. Immunosuppression and Infection Risk 

Infectious diseases represent a major threat to patients’ safety in MM. A Swedish population-based study conducted in 9253 MM patients between 1988 and 2004 reported a 7-fold increased risk of developing bacterial infections and 10-fold increased risk for viral infections compared to population-based matched control subjects. Similarly, the 3-year risk of infection-related death was 12.2% vs. 2.2% significantly higher in MM patients compared to controls [61].

Patients with plasma cell dyscrasia have a dysfunctional immune system secondary to immunoparesis and cellular immune defects, including the impaired function of dendritic cells, and B and T lymphocytes [62,63]. The suppression of polyclonal immunoglobulin is observed in up to 60% of relapsed MM patients and correlates with tumor burden. Importantly, immunoparesis has a negative prognostic impact on overall survival (OS) with 3-year OS estimates of 36% for patients with deep immunoparesis (defined as a relative difference between polyclonal immunoglobulin and corresponding lower normal limit < 50%) compared to 46% for patients without [63]. In addition, anti-MM agents further aggravate immunosuppression by inducing leucopenia, impairing virus-specific cytotoxic T lymphocytes response, or depleting NK cells. In particular, PI and daratumumab induce the viral reactivation of herpes [64,65,66]. Importantly, increasing lines of therapy (>4) are associated with a sevenfold increased risk of infection [67].

In addition to a proactive management of infections with antibiotic, antiviral, and antifungal agents as needed, prophylactic strategies are also strongly recommended. Based on the EHA-ESMO guidelines, patients should be vaccinated against influenza A and B, Streptococcus pneumonia, and Hemophilus influenzae [68]. In general, only live virus vaccines should be avoided in MM patients. If treated with PI or daratumumab, MM patients should receive antivirals, such as valaciclovir or acyclovir [69,70]. In addition, patients could be offered the varicella zoster virus (VZV) recombinant vaccine, Shingrix [68,71]. This vaccine has been recently approved in adults aged 50 years or older and all adults (≥18) at risk for VZV reactivation. The administration schedule consists of two doses given 2 to 6 months apart. In a phase 3 trial in patients with hematological malignancies, 80% of the people receiving the vaccine had a humoral response, which persisted for one year, compared to 0,8% in the placebo group [72]. Similarly, a study assessing MM patients undergoing active treatment reported seroconversion in 81% and 89% of the patients receiving the first and second dose of Shingrix, respectively [73]. MM patients may also profit from immunoglobulin replacement therapy (IgRT). Although the routine prophylaxis with IgRT is not recommended, it should be considered in case of low IgG levels (<400–500 mg/dL) and at least two severe infections needing hospitalization in the past 12 months [69]. The recommendation is based on a meta-analysis of nine clinical trials of IgRT in chronic lymphocytic leukemia and MM, which showed a significant reduction in the rate of major infections with no survival benefit [74]. IgRT is commonly administrated as a monthly intravenous infusion, requiring hospital stay and leading to concentration peaks. An alternative strategy is the subcutaneous (sc) application, whose advantages rely on the possibility of home therapy, the stable steady state concentrations, and the mainly local side effects [75]. Vacca et al. reported the results of a small, randomized trial in MM patients, both newly diagnosed and relapsed, with secondary hypogammaglobulinemia treated for six months with sc IgRT or not. Treatment with sc IgRT protected MM patients from severe infections and reduced antibiotic use, thus improving quality of life and treatment adherence [76].

### SARS-CoV-2 Infection and Vaccine

In the last 18 months, the management of cancer patients has proved quite challenging due to the rapid and broad diffusion of a new pathogenic agent, SARS-CoV-2. During the pandemic, diagnostic delays have been frequent, and treatments have often been modified to privilege oral or subcutaneous drug combinations to reduce hospital access [77]. Data currently available on coronavirus disease 2019 (COVID-19) in MM patients is still limited but constantly updated.

A retrospective case series of 167 MM patients hospitalized with COVID-19 was reported from the Spanish Myeloma Collaborative Group. Patients had a median age of 71 years, and two thirds of them had at least one comorbidity. More than 75% of the patients had a moderate to severe clinical presentation and up to 21% required noninvasive ventilation. The inpatient mortality rate was 34% vs. 23% higher in MM patients compared to an age-/sex-matched noncancer control group. Age > 65 years, active/progressive disease, and renal disease were independent prognostic factors for mortality in this patient cohort [78]. Similarly, a retrospective study of the International Myeloma Society (IMS) including 650 patients from 10 different countries confirmed the high mortality rate in hospitalized patients with MM and COVID-19 infections, ranging from 27% in Germany and Italy to 57% in the United Kingdom. The multivariate analysis identified age, high-risk cytogenetic (del 17p, t(4;14), amp 1q or t(14;16)), active disease, and renal failure (CrCl < 40 mL/min) as the independent predictors of death [79].

Considering the higher mortality due to COVID-19 of MM patients compared to non-MM subjects, measures to prevent SARS-CoV-2 infection are of critical importance and include social distancing, wearing masks, and vaccination. The IMS recommends that all patients with MM, smoldering MM, and MGUS should be candidates for the SARS-CoV-2 vaccine, based on approval from local regulatory authorities [80]. However, in contrast to the general population, in MM, the ability of SARS-CoV-2 vaccines to generate an effective immune response might be impaired [81]. Terpos et al. reported an antibody response (neutralizing antibody >50% 21 days after the first dose) to mRNA vaccine in elderly MM patients of 8.3% compared with 20.2% in the control group [82]. Similarly, Pimpinelli et al. observed seroprotection (titer of neutralizing anti-SARS-CoV-2 IgG > 15 AU/mL) in 78.6% of MM patients receiving the BNT162b2 mRNA vaccine compared to 100% of controls. Interestingly, treatment with daratumumab was associated with a lower vaccine response [83]. These studies raise several questions, including the need for additional boosters, antibody titer monitoring, and maintaining masks and social distancing for MM patients at risk of COVID-19. Due to the rapidly evolving situation, treatment decisions should follow the most updated guidelines (Table 2).

## 6. Drug-Specific Non-Hematological Adverse Events of Special Interest

The recent approval of several new agents has significantly expanded the therapeutic armamentarium against MM. Recommended agents in the RR setting include IMiDs, PI, targeted therapies, and monoclonal antibodies, all with unique toxicity profiles (Table 3) [7]. Preserving quality of life is a priority for most patients with RRMM and choice of treatment should take into consideration the accumulated toxicity and morbidity. In addition, treatment compliance depends on the timely management of side effects, and an increased awareness of the toxicity profiles of next-generation anti-MM agents is therefore critical to therapy success.

### 6.1. Peripheral Neuropathy

Neurological disorders in MM are either a direct consequence of the disease or induced by specific treatments. The deposition of amyloid, cytokine-mediated nerve damage, or direct nerve compression may all contribute to MM-associated peripheral neuropathy (PN). Neurotoxic anti-MM agents include chemotherapy, IMIDs (especially thalidomide), and PI [93]. Bortezomib, in particular, is frequently associated with severe PN in the RR setting with up to 35% of patients reporting treatment-emergent PN and 8–13% with severe PN [86,94]. In contrast, the incidence of PN with carfilzomib and ixazomib is lower, below 10% [25,87].

The pathogenesis of bortezomib-induced PN (BIPN) relies on mitochondria and endoplasmic reticulum damage, oxidative stress, and inflammation, leading predominantly to axonal injury of the dorsal root ganglia [95,96]. In addition, rare cases of demyelinating polyradiculoneuritis have also been reported [97,98]. In contrast, MM-induced PN depends mainly on segmental demyelination and axonal degeneration due to plasma cell infiltration and amyloid deposition within the nerve [99,100].

BIPN typically manifests as a sensory axonopathy characterized by symmetric paresthesia, burning sensation and neuropathic pain in stocking and glove distribution. Among the risk factors for BIPN, pre-existing neuropathy plays an important role. In the analysis of Richardson et al., more than 80% of RRMM patients suffer from PN at the start of treatment, which is further exacerbated by exposure to bortezomib [94]. BIPN also depends on the cumulative dose and schedule of administration, whereas the mode of administration is not relevant [101]. Since no curative treatment is available for BIPN, prevention represents a key strategy to limit toxicity. Careful assessment of patients for baseline neuropathy by means of specific questionnaires, such as the Functional Assessment of Cancer Therapy Scale/Gynecologic Oncology Group-Neurotoxicity (FACT/GOG-Ntx), and neurological examination is mandatory before treatment initiation [94]. In case of grade 1 PNP, dose reduction and/or switch to once-weekly administration is recommended and may lead to improvement or resolution in the majority of patients [94,102,103]. In addition to dose and schedule adjustments, alleviation of symptoms may be achieved with multi-B vitamin supplements and neuroleptic medications, such as gabapentin, pregabalin, amitriptyline and duloxetine [93]. Additional treatment strategies include topical treatments with lidocaine or capsaicin patches [93,104], as well as acupuncture, which significantly improves patient-reported sensory symptoms and motor disfunction due to BIPN [105].

### 6.2. Venous Thromboembolism

MM patients are at risk of developing venous thromboembolism (VTE) due to hyperviscosity, tumor-intrinsic properties as well as treatment [106,107]. In particular, thalidomide and lenalidomide-based combination strategies are associated with an increased thrombotic risk, with up to 15% of Caucasian and up to 8% of Asian RRMM patients experiencing VTE events [108,109,110,111,112].

VTE prophylaxis with low-dose acetylsalicylic acid or low molecular weight heparin (LMWH) is compulsory in IMiDs-treated MM at high risk for VTE. Risk factors include personal or family history of VTE, smoking history, performance status > 1, use of oral contraceptives and, importantly, concurrent use of epoetin or dexamethasone [113]. Prophylactic anticoagulation therapy is therefore not recommended in MM patients receiving single-agent IMID treatment as maintenance strategy in the absence of risk factors for VTE [114]. In contrast, patients treated with IMIDs and dexamethasone should receive VTE prophylaxis and treatment choice is based on risk stratification according to IMWG guidelines [113,115]. Recently, based on the results of the IMPEDE VTE study a new algorithm with three risk categories has been proposed [116]. The IMPEDE VTE prediction tool comprises nine variables: use of IMIDs, ESAs, and/or dexamethasone/doxorubicin; body mass index; pelvic, hip or femur fracture; race; VTE history; central venous catheter; and ongoing thromboprophylaxis. Patients are classified as having low, intermediate or high risk of developing VTE within six months of treatment initiation if the overall score is ≤ 3, 4–7 or ≥8, respectively. Despite validation in newly diagnosed MM patients, the algorithm may effectively identify patients at high-risk for VTE also in the RR setting [117].

2 to 9% of RRMM patients treated with lenalidomide- and pomalidomide-based combinations experience VTE events during thromboprophylaxis, with the main risk factors being previous VTE and age [18,111,118,119,120]. Therefore, ongoing studies are evaluating the risk/benefit profile of novel oral anticoagulants. In a pilot study assessing primary prevention of VTE in MM patients treated with lenalidomide or pomalidomide, apixaban was well-tolerated without major hemorrhage events and effectively protected against VTE, stroke and myocardial infarction in the 6-month follow-up [121,122].

### 6.3. Cardiotoxicity 

In MM patients advanced age and pre-existing risk factors, such as diabetes and hypertension, increase the risk for cardiovascular (CV) disease, which is further exacerbated by MM-associated factors, such as amyloidosis and renal failure, and anti-MM treatment, in particular radiotherapy to sternal lesions, chemotherapy, such as doxorubicin, and irreversible proteasome inhibitors, in particular carfilzomib [123]. CV complications include hypertension, peripheral edema, dyspnea, ischemia, and heart failure. They are often treatment-limiting events and adversely impact survival of MM patients [8].

As recently shown in a prospective study in relapsed MM, CV adverse events are common in patients treated with carfilzomib, occurring as grade 3 events or higher in more than half of the patients within the first three months of treatment. In contrast, bortezomib and ixazomib are associated to a lower rate of CV adverse events [8,124]. Importantly, a recent analysis of real-world data suggests a higher incidence of hypertension in patients treated with carfilzomib compared to that reported in clinical trials (60% vs. 18%) [125,126]. Risk factors for CV toxicity include baseline elevated pBNP levels and higher doses of carfilzomib (≥45 mg/m^2^), [127] whereas treatment line and combination strategies are not significantly affecting CV risk [128].

Recommendations to reduce the incidence of CV adverse events include the treatment of baseline cardiovascular risk factors, the identification of patients at risk for CV disease, who require more intense follow-ups, and the regular monitoring of blood pressure to allow a timely start of antihypertensive agents [129]. In case of carfilzomib-induced CV adverse events, a dose reduction or, eventually, discontinuation may be indicated based on severity. Considering the widespread use of carfilzomib, guidelines on the management of carfilzomib-induced CV events have been recently published by the European Myeloma Network and the Italian Society of Arterial Hypertension [129].

### 6.4. Gastrointestinal Toxicity 

Gastrointestinal (GI) toxicity is one of the most frequent side effects of anti-MM agents. Up to 60% of RRMM patients treated with IMiDs and PIs suffer from nausea, vomiting, diarrhea, and loss of appetite, which may be further exacerbated by opioid-based analgesia, lifestyle, and diet. Most symptoms are mild to moderate and are successfully treated with supportive therapies [130]. However, if left untreated, severe GI manifestations may have serious consequences such as abdominalgia, weight loss, and electrolyte imbalance. Of note, IMIDs are often associated to late onset diarrhea. Lenalidomide, in particular, may result in chronic diarrhea due to bile acid malabsorption [131]. Retrospective studies suggest that treatment with bile acid sequestrant, such as colesevelam, and/or reduction in dietary fat intake significantly improve stool consistency and frequency, thus enabling the continuation of anti-MM therapy [132]. A phase 2 study to assess the efficacy of colesevelam in MM patients on lenalidomide maintenance is currently ongoing (NCT03767257).

Nausea and vomiting are frequently observed during treatment with selinexor, an oral inhibitor of the nuclear export protein exportin 1 [27]. Based on a recent study reviewing data from more than 400 patients enrolled in clinical trials, nausea occurred in 68% and vomiting in 37% of the patients [133]. The incidence of GI symptoms is at its highest within the first two weeks of treatment and drops thereafter. The median time to onset is three days, and GI toxicity may last for up to three weeks if not treated. Nausea and vomiting are reversible upon drug discontinuation, and the use of supportive care such as dexamethasone, 5-HT3 antagonists, and neurokinin 1 receptor antagonists effectively mitigates nausea. Therefore, antiemetic prophylaxis is strongly recommended to improve tolerance to selinexor and ensure treatment adherence [134].

In addition to drug toxicity, immunoglobulin-derived light chain (AL) amyloidosis should also be considered in the differential diagnosis of MM patients presenting with GI symptoms [135]. Indeed, amyloid deposition in the autonomic nervous system, liver, and GI tract may lead to bowel dysmotility, gastroparesis with early satiety, macroglossia, hepatomegaly, GI bleeding, malabsorption, diarrhea, and weight loss in up to 10% of MM patients [136,137]. GI amyloidosis is confirmed by the identification of amyloid deposits with congo red stain in biopsy specimens from bone marrow, subcutaneous fat, or affected organs. Less invasive approaches based on functional imaging such as 18F-Florbetaben PET scans are under investigation [138].

### 6.5. Ocular Toxicity

Treatment-related adverse events involving the eye were until recently extremely rare in MM patients [139,140]. However, the approval of belantamab mafodotin (belamaf; GSK2857916) for the treatment of RRMM in July 2020 drastically increased the rate of ocular toxicity [91]. Belamaf is a B-cell maturation antigen (BCMA)-targeting antibody conjugated to the cytotoxic agent monomethyl auristatin F (MMAF), which is typically associated with keratopathy. Indeed, up to 75% of the patients treated with Belamaf develop microcyst-like epithelial changes (MEC) of the cornea and superficial punctate keratopathy [141,142]. Symptoms include blurred vision and dry eyes, but corneal lesions may also remain asymptomatic. Diagnosis is based on slit lamp exam and Snellen visual acuity testing [143]. Ocular toxicity increases with higher doses and history of dry eyes but is reversible upon drug discontinuation. Regular monitoring before treatment initiation and at every cycle is recommended. In case of ocular toxicity, a dose reduction or treatment interruption for severe disease is recommended [143].

## 7. Discussion

By preventing disease and treatment-related complications, supportive care significantly contributes to improved outcomes in MM. However, physicians still face several challenges in delivering optimal therapy. For example, organizational hurdles and patients´ preference may jeopardize guidelines´ adherence. A recent analysis of the “Surveillance, Epidemiology, and End Results-Medicare“ database (https://seer.cancer.gov/, accessed on 4 October 2021) showed that only 64% of older adults with active MM received bone-modifying agents, 52% an influenza vaccination, and less than 50% antiviral prophylaxis during treatment with PI [144]. Similarly, a French study reported very low rates of vaccination for influenza (28%) in MM patients, with less than 1% of the patients receiving all three recommended vaccines (influenza, Streptococcus pneumoniae, and Hemophilus influenzae) [145]. The frequent risk factors for the underutilization of recommended care are older age, higher comorbidity burden, and care in the community setting. Specific interventions targeting these subgroups of patients, such as the integration of primary care providers and clinical decision support systems, are required to implement quality of care in MM.

Similarly, the discrepancy between clinical trials and real-world MM patients questions the safety data of novel agents, thus posing an additional challenge for health care providers. Indeed, up to 70% of patients with RRMM do not meet the eligibility criteria of phase 3 studies because of comorbidities such as renal and cardiovascular disease [146]. Recently, Chavda et al. observed a higher incidence of hypertension in unselected MM patients treated with carfilzomib compared to clinical trial data (60% vs. 18%) [125,126]. Poor tolerability of pomalidomide requiring frequent dose reduction has been reported in elderly RRMM patients with poor performance status [147]. Several initiatives have been proposed to broaden the standard inclusion/exclusion criteria of clinical trials, therefore ensuring a more representative real-world MM patient population [148].

Research progress in MM is leading to a dramatic increase in the number of new drugs and the complexity of combination regimens, demanding constant updates on their indications, mechanisms of action, and safety profiles. T-cell engaging treatments, such as bispecific antibodies (T-BsAbs) and chimeric antigen receptor (CAR) T cells, have revolutionized the treatment of hematological cancers, including MM [149,150,151]. In addition to cytopenia and infection, cytokine release syndrome (CRS) and neurotoxicity represent common and potentially fatal adverse events of these agents [152,153]. CRS is a systemic inflammatory response occurring within few weeks of drug administration due to a massive T cell stimulation with consequent cytokine storm (including IL-6, IL-2R, IL-10, IFN-γ, and TNF-α). CRS may be mild with flu-like symptoms, but few patients may develop severe life-threatening manifestations including circulatory shock, disseminated intravascular coagulation, and multi-organ system failure. The monoclonal antibody against IL-6, tocilizumab, represents the treatment of choice in case of CRS. Frequency and severity of CRS depend on the therapeutic agent. In patients treated with T-BsAbs the incidence of CRS ranges from 40 to 70%, and intensity is usually mild to moderate [151,154]. On the contrary, treatment with CAR-T cells is complicated by CRS in almost 90% of the patients, and 7 to 30% may experience grade 3 or worse toxicity [149,155]. Similarly, neurotoxicity has been mainly reported in CAR-T cell trials with an overall frequency of 36%. Two syndromes have been recognized: CAR-T cell-related encephalopathy syndrome (CRES) and immune effector cell-associated neurotoxicity syndrome (ICANS). CRES and ICANS are probably related to the disruption of the blood–brain barrier, enabling lymphocyte and cytokine infiltration in the cerebrospinal fluid. Symptoms range from confusion to paresis and seizures, and treatment is based on intravenous steroids [156]. Despite their recent FDA approval, the complexity of the treatment and the spectrum of therapy-related complications requires an interdisciplinary team of hematologists, neurologists, and intensive care physicians and therefore limits their use only to selected medical facilities [156]. In addition, late-onset side effects may become increasingly relevant due to the long-lasting remissions and improved survival rates of MM patients. For instance, secondary malignancies have been described in RRMM patients receiving lenalidomide, and spontaneous vertebral fractures have been observed in breast cancer patients after long-term treatment with denosumab [157,158].

## 8. Conclusions

In conclusion, optimal care of the disease and treatment-related complications has a pivotal role in MM, not only to improve health-related quality of life but also to prolong patients´ survival. An awareness of drug toxicity allows for early diagnosis and intervention, thus supporting longer treatment exposure and better response rates.

## Figures and Tables

**Table 1:** Overview of renal response in phase 3 clinical trials in RRMM with RI.

Study	Regimen	Baseline Renal Function	Renal Response	PFS	OS	Discontinuation Due to AEs
Retrospective analysis [21]	Pegylated liposomal doxorubicin and bortezomib (*n* = 95) vs. bortezomib (*n* = 98)	CrCl 30–60 mL/min	Improvement	13.6 vs. 6.9 months	NA	4.3 vs. 2.1% *
Subgroup analysis of the MM-009 and MM-010 trials [18]	Lenalidomide and dexamethasone (*n* = 353)	CrCl < 60 mL/min	70% ^a^	7.8 months (CrCl < 30) 9.5 months (CrCl 30–59)	18.4 months (CrCl < 30) 29 months (CrCl 30–59)	38% (CrCl < 30) 18% (CrCl 30–59)
Retrospective analysis of the MM-003 trial [22]	Pomalidomide and low-dose dexamethasone (*n* = 93) vs. high dose dexamethasone (*n* = 56)	CrCl 30–60 mL/min	42% vs. 47% ^a^	4 vs. 1.9 months	10.4 vs. 4.9 months	13 vs. 11%
Post hoc exploratory analysis of the Endeavor trial [9]	Carfilzomib and dexamethasone (*n* = 85) vs. bortezomib and dexamethasone (*n* = 99)	CrCl 15–50 mL/min	15.3% vs. 14.1% ^b^	14.9 vs. 6.5 months	42.1 vs. 23.7 months	31.8% vs. 23.7%
Prespecified subgroup analysis of the ICARIA-MM trial [20]	Isatuximab, pomalidomide and dexamethasone (*n* = 55) vs. pomalidomide and dexamethasone (*n* = 49)	eGFR 30–60 mL/min/1.73 m^2^	71.9% vs. 38.1% ^c^	9.5 vs. 3.7 months	NR vs. 11.6 months	11.1% vs. 14.9%

Abbreviations: RRMM, relapsed/refractory multiple myeloma; RI, renal impairment; PFS, progression-free survival; OS, overall survival; AEs, adverse events; eGFR, estimated glomerular filtration rate; CrCl, creatinine clearance; mo, months; NR, not reached; NA, not available. ^a^ CrCl 30–59 to CrCl ≥ 60 mL/min. ^b^ CrCl < 60 to CrCl ≥ 60 mL/min. ^c^ eGFR < 50 to eGFR ≥ 60 mL/min/1.73 m^2^. * Death due to AEs.

**Table 2:** Specific recommendations and continuously updated resources on COVID-19 in cancer patients.

Association	Website
European Hematology Association (EHA)	https://ehaweb.org/covid-19/covid-19-recommendations/recommendations-for-specific-hematologic-malignancies/ (accessed on 4 October 2021)
American Association of Hematology (ASH)	https://www.hematology.org/covid-19 (accessed on 4 October 2021)
European Society of Medical Oncology (ESMO)	https://www.esmo.org/covid-19-and-cancer (accessed on 4 October 2021)
American Society of Clinical Oncology (ASCO)	https://www.asco.org/asco-coronavirus-information (accessed on 4 October 2021)
National Cancer Institute (NCI-NIH)	Home|NIH COVID-19 Research (accessed on 4 October 2021 )
National Comprehensive Cancer Network (NCCN)	https://www.nccn.org/covid-19 (accessed on 4 October 2021)

**Table 3:** Extra-hematological Grade 3/4 adverse events of specific interest in RRMM.

Drug (Combination Agents)	PN	VTE	C	O	GI	I
Lenalidomide (dexamethasone) [84]	1.7%	14.7%	2.8%		9.6%	21.4%
Pomalidomide (dexamethasone) [85]		1%	6.3%		2.3%	34%
Bortezomib [86]	8%		6%		17%	
Carfilzomib (dexamethasone) [25]	1%		15%		6.9%	2%
Ixazomib (lenalidomide, dexamethasone) [87]	2%	3%	3%		9.4%	1%
Elotuzumab (lenalidomide, dexamethasone) [88]			1%		6%	1%
Daratumumab (lenalidomide, dexamethasone) [89]			4%		8.9%	8.9%
Isatuximab [90]						7%
Belantamab [91]			6%	21–27%	3%	4–11%
Selinexor [27]			5.6%	2%	25%	10.5%
Panobinostat (bortezomib, dexamethasone) [92]	17.5%		7.3%		42%	16%

Abbreviations: PN: peripheral neuropathy; VTE: venous thromboembolism; C: cardiac toxicity; O: ocular toxicity; GI: gastrointestinal toxicity; I: infections.

## References

[1] Cowan A.J., Allen C., Barac A., Basaleem H., Bensenor I., Curado M.P., Foreman K., Gupta R., Harvey J., Hosgood H.D. (2018). Global Burden of Multiple Myeloma: A Systematic Analysis for the Global Burden of Disease Study 2016. JAMA Oncol..

[2] Mai E.K., Haas E.M., Lucke S., Lopprich M., Kunz C., Pritsch M., Knaup-Gregori P., Raab M.S., Schlenzka J., Bertsch U. (2018). A systematic classification of death causes in multiple myeloma. Blood Cancer J..

[3] Nishimura K.K., Barlogie B., van Rhee F., Zangari M., Walker B.A., Rosenthal A., Schinke C., Thanendrarajan S., Davies F.E., Hoering A. (2020). Long-term outcomes after autologous stem cell transplantation for multiple myeloma. Blood Adv..

[4] Langseth O.O., Myklebust T.A., Johannesen T.B., Hjertner O., Waage A. (2020). Incidence and survival of multiple myeloma: A population-based study of 10,524 patients diagnosed 1982–2017. Br. J. Haematol..

[5] Thorsteinsdottir S., Dickman P.W., Landgren O., Blimark C., Hultcrantz M., Turesson I., Bjorkholm M., Kristinsson S.Y. (2018). Dramatically improved survival in multiple myeloma patients in the recent decade: Results from a Swedish population-based study. Haematologica.

[6] Majithia N., Vincent Rajkumar S., Lacy M.Q., Buadi F.K., Dispenzieri A., Gertz M.A., Hayman S.R., Dingli D., Kapoor P., Hwa L. (2015). Outcomes of primary refractory multiple myeloma and the impact of novel therapies. Am. J. Hematol..

[7] Bazarbachi A.H., Al Hamed R., Malard F., Harousseau J.L., Mohty M. (2019). Relapsed refractory multiple myeloma: A comprehensive overview. Leukemia.

[8] Cornell R.F., Ky B., Weiss B.M., Dahm C.N., Gupta D.K., Du L., Carver J.R., Cohen A.D., Engelhardt B.G., Garfall A.L. (2019). Prospective Study of Cardiac Events During Proteasome Inhibitor Therapy for Relapsed Multiple Myeloma. J. Clin. Oncol..

[9] Dimopoulos M., Siegel D., White D.J., Boccia R., Iskander K.S., Yang Z., Kimball A.S., Mezzi K., Ludwig H., Niesvizky R. (2019). Carfilzomib vs bortezomib in patients with multiple myeloma and renal failure: A subgroup analysis of ENDEAVOR. Blood.

[10] Mohyuddin G.R., Koehn K., Shune L., Aziz M., Abdallah A.O., McClune B., Ganguly S., McGuirk J., Kambhampati S. (2021). Renal insufficiency in multiple myeloma: A systematic review and meta-analysis of all randomized trials from 2005–2019. Leuk. Lymphoma.

[11] Niesvizky R., Naib T., Christos P.J., Jayabalan D., Furst J.R., Jalbrzikowski J., Zafar F., Mark T., Lent R., Pearse R.N. (2007). Lenalidomide-induced myelosuppression is associated with renal dysfunction: Adverse events evaluation of treatment-naive patients undergoing front-line lenalidomide and dexamethasone therapy. Br. J. Haematol..

[12] Dimopoulos M.A., Sonneveld P., Leung N., Merlini G., Ludwig H., Kastritis E., Goldschmidt H., Joshua D., Orlowski R.Z., Powles R. (2016). International Myeloma Working Group Recommendations for the Diagnosis and Management of Myeloma-Related Renal Impairment. J. Clin. Oncol..

[13] Bridoux F., Leung N., Belmouaz M., Royal V., Ronco P., Nasr S.H., Fermand J.P. (2021). International Kidney and Monoclonal Gammopathy Research Group. Management of acute kidney injury in symptomatic multiple myeloma. Kidney Int..

[14] Bridoux F., Carron P.L., Pegourie B., Alamartine E., Augeul-Meunier K., Karras A., Joly B., Peraldi M.N., Arnulf B., Vigneau C. (2017). Effect of High-Cutoff Hemodialysis vs Conventional Hemodialysis on Hemodialysis Independence Among Patients With Myeloma Cast Nephropathy: A Randomized Clinical Trial. JAMA.

[15] Hutchison C.A., Cockwell P., Moroz V., Bradwell A.R., Fifer L., Gillmore J.D., Jesky M.D., Storr M., Wessels J., Winearls C.G. (2019). High cutoff versus high-flux haemodialysis for myeloma cast nephropathy in patients receiving bortezomib-based chemotherapy (EuLITE): A phase 2 randomised controlled trial. Lancet Haematol..

[16] Ponisch W., Moll B., Bourgeois M., Andrea M., Schliwa T., Heyn S., Schmalfeld M., Edelmann T., Becker C., Hoffmann F.A. (2013). Bendamustine and prednisone in combination with bortezomib (BPV) in the treatment of patients with relapsed or refractory multiple myeloma and light chain-induced renal failure. J. Cancer Res. Clin. Oncol..

[17] Tosi P., Zamagni E., Cellini C., Cangini D., Tacchetti P., Tura S., Baccarani M., Cavo M. (2004). Thalidomide alone or in combination with dexamethasone in patients with advanced, relapsed or refractory multiple myeloma and renal failure. Eur. J. Haematol..

[18] Dimopoulos M., Alegre A., Stadtmauer E.A., Goldschmidt H., Zonder J.A., de Castro C.M., Masliak Z., Reece D., Olesnyckyj M., Yu Z. (2010). The efficacy and safety of lenalidomide plus dexamethasone in relapsed and/or refractory multiple myeloma patients with impaired renal function. Cancer.

[19] Kumar S., Fu A., Niesvizky R., Jagannath S., Boccia R., Raje N. (2021). Renal response in real-world carfilzomib- vs bortezomib-treated patients with relapsed or refractory multiple myeloma. Blood Adv..

[20] Dimopoulos M.A., Leleu X., Moreau P., Richardson P.G., Liberati A.M., Harrison S.J., Miles Prince H., Ocio E.M., Assadourian S., Campana F. (2021). Isatuximab plus pomalidomide and dexamethasone in relapsed/refractory multiple myeloma patients with renal impairment: ICARIA-MM subgroup analysis. Leukemia.

[21] Blade J., Sonneveld P., San Miguel J.F., Sutherland H.J., Hajek R., Nagler A., Spencer A., Robak T., Cibeira M.T., Zhuang S.H. (2008). Pegylated liposomal doxorubicin plus bortezomib in relapsed or refractory multiple myeloma: Efficacy and safety in patients with renal function impairment. Clin. Lymphoma Myeloma.

[22] Weisel K.C., Dimopoulos M.A., Moreau P., Lacy M.Q., Song K.W., Delforge M., Karlin L., Goldschmidt H., Banos A., Oriol A. (2016). Analysis of renal impairment in MM-003, a phase III study of pomalidomide + low-dose dexamethasone versus high-dose dexamethasone in refractory or relapsed and refractory multiple myeloma. Haematologica.

[23] Liu L., Yu Z., Cheng H., Mao X., Sui W., Deng S., Wei X., Lv J., Du C., Xu J. (2020). Multiple myeloma hinders erythropoiesis and causes anaemia owing to high levels of CCL3 in the bone marrow microenvironment. Sci. Rep..

[24] Ludwig H., Fritz E., Kotzmann H., Hocker P., Gisslinger H., Barnas U. (1990). Erythropoietin treatment of anemia associated with multiple myeloma. N. Engl. J. Med..

[25] Dimopoulos M.A., Moreau P., Palumbo A., Joshua D., Pour L., Hajek R., Facon T., Ludwig H., Oriol A., Goldschmidt H. (2016). Carfilzomib and dexamethasone versus bortezomib and dexamethasone for patients with relapsed or refractory multiple myeloma (ENDEAVOR): A randomised, phase 3, open-label, multicentre study. Lancet Oncol..

[26] Palumbo A., Chanan-Khan A., Weisel K., Nooka A.K., Masszi T., Beksac M., Spicka I., Hungria V., Munder M., Mateos M.V. (2016). Daratumumab, Bortezomib, and Dexamethasone for Multiple Myeloma. N. Engl. J. Med..

[27] Chari A., Vogl D.T., Gavriatopoulou M., Nooka A.K., Yee A.J., Huff C.A., Moreau P., Dingli D., Cole C., Lonial S. (2019). Oral Selinexor-Dexamethasone for Triple-Class Refractory Multiple Myeloma. N. Engl. J. Med..

[28] Kumar S.K., Callander N.S., Adekola K., Anderson L., Baljevic M., Campagnaro E., Castillo J.J., Chandler J.C., Costello C., Efebera Y. (2020). Multiple Myeloma, Version 3.2021, NCCN Clinical Practice Guidelines in Oncology. J. Natl. Compr. Cancer Netw..

[29] Horvath-Puho E., Suttorp M.M., Frederiksen H., Hoekstra T., Dekkers O.M., Pedersen L., Cannegieter S.C., Dekker F.W., Sorensen H.T. (2018). Erythropoiesis-stimulating agents and cardiovascular events in patients with myelodysplastic syndrome and multiple myeloma. Clin. Epidemiol..

[30] Anaissie E.J., Coleman E.A., Goodwin J.A., Kennedy R.L., Lockhart K.D., Stewart C.B., Coon S.K., Bailey C., Barlogie B. (2012). Prophylactic recombinant erythropoietin therapy and thalidomide are predictors of venous thromboembolism in patients with multiple myeloma: Limited effectiveness of thromboprophylaxis. Cancer.

[31] Knight R., DeLap R.J., Zeldis J.B. (2006). Lenalidomide and venous thrombosis in multiple myeloma. N. Engl. J. Med..

[32] Ludwig H., Miguel J.S., Dimopoulos M.A., Palumbo A., Garcia Sanz R., Powles R., Lentzsch S., Ming Chen W., Hou J., Jurczyszyn A. (2014). International Myeloma Working Group recommendations for global myeloma care. Leukemia.

[33] Verma A., Suragani R.N., Aluri S., Shah N., Bhagat T.D., Alexander M.J., Komrokji R., Kumar R. (2020). Biological basis for efficacy of activin receptor ligand traps in myelodysplastic syndromes. J. Clin. Investig..

[34] Abdulkadyrov K.M., Salogub G.N., Khuazheva N.K., Sherman M.L., Laadem A., Barger R., Knight R., Srinivasan S., Terpos E. (2014). Sotatercept in patients with osteolytic lesions of multiple myeloma. Br. J. Haematol..

[35] Fenaux P., Platzbecker U., Mufti G.J., Garcia-Manero G., Buckstein R., Santini V., Diez-Campelo M., Finelli C., Cazzola M., Ilhan O. (2020). Luspatercept in Patients with Lower-Risk Myelodysplastic Syndromes. N. Engl. J. Med..

[36] Belot A., Gourbeyre O., Fay A., Palin A., Besson-Fournier C., Latour C., Hopkins C.R., Tidmarsh G.F., Coppin H., Roth M.P. (2020). LJ000328, a novel ALK2/3 kinase inhibitor, represses hepcidin and significantly improves the phenotype of IRIDA. Haematologica.

[37] Kanamori Y., Sugiyama M., Hashimoto O., Murakami M., Matsui T., Funaba M. (2016). Regulation of hepcidin expression by inflammation-induced activin B. Sci. Rep..

[38] Terpos E., Kanellias N., Moulopoulos L., Christoulas D., Gavriatopoulou M., Migkou M., Bagratuni T., Koutoulidis V., Kastritis E., Dimopoulos M. (2019). Incidence of Skeletal-Related Events at Diagnosis and at the Time of First Relapse in 463 Patients with Multiple Myeloma Who Received First Line Treatment in a Single Center. Clin. Lymphoma Myeloma Leuk..

[39] Terpos E., Zamagni E., Lentzsch S., Drake M.T., Garcia-Sanz R., Abildgaard N., Ntanasis-Stathopoulos I., Schjesvold F., de la Rubia J., Kyriakou C. (2021). Treatment of multiple myeloma-related bone disease: Recommendations from the Bone Working Group of the International Myeloma Working Group. Lancet Oncol..

[40] Morgan G.J., Davies F.E., Gregory W.M., Bell S.E., Szubert A.J., Cook G., Drayson M.T., Owen R.G., Ross F.M., Jackson G.H. (2013). Long-term follow-up of MRC Myeloma IX trial: Survival outcomes with bisphosphonate and thalidomide treatment. Clin. Cancer Res..

[41] Morgan G.J., Davies F.E., Gregory W.M., Cocks K., Bell S.E., Szubert A.J., Navarro-Coy N., Drayson M.T., Owen R.G., Feyler S. (2010). First-line treatment with zoledronic acid as compared with clodronic acid in multiple myeloma (MRC Myeloma IX): A randomised controlled trial. Lancet.

[42] Anderson K., Ismaila N., Flynn P.J., Halabi S., Jagannath S., Ogaily M.S., Omel J., Raje N., Roodman G.D., Yee G.C. (2018). Role of Bone-Modifying Agents in Multiple Myeloma: American Society of Clinical Oncology Clinical Practice Guideline Update. J. Clin. Oncol..

[43] Garcia-Sanz R., Oriol A., Moreno M.J., de la Rubia J., Payer A.R., Hernandez M.T., Palomera L., Teruel A.I., Blanchard M.J., Gironella M. (2015). Zoledronic acid as compared with observation in multiple myeloma patients at biochemical relapse: Results of the randomized AZABACHE Spanish trial. Haematologica.

[44] Mateos M.V., Fink L., Koneswaran N., Intorcia M., Giannopoulou C., Niepel D., Cavo M. (2020). Bone complications in patients with multiple myeloma in five European countries: A retrospective patient chart review. BMC Cancer.

[45] Raje N., Terpos E., Willenbacher W., Shimizu K., Garcia-Sanz R., Durie B., Legiec W., Krejci M., Laribi K., Zhu L. (2018). Denosumab versus zoledronic acid in bone disease treatment of newly diagnosed multiple myeloma: An international, double-blind, double-dummy, randomised, controlled, phase 3 study. Lancet Oncol..

[46] Gupta A., Wang P., Ali S.A., Rajkumar S.V., Gyawali B., Overton H.N., Makary M.A. (2020). Use of Bone-Modifying Agents Among Medicare Beneficiaries with Multiple Myeloma. JAMA Oncol..

[47] Vij R., Horvath N., Spencer A., Taylor K., Vadhan-Raj S., Vescio R., Smith J., Qian Y., Yeh H., Jun S. (2009). An open-label, phase 2 trial of denosumab in the treatment of relapsed or plateau-phase multiple myeloma. Am. J. Hematol..

[48] Major P., Lortholary A., Hon J., Abdi E., Mills G., Menssen H.D., Yunus F., Bell R., Body J., Quebe-Fehling E. (2001). Zoledronic acid is superior to pamidronate in the treatment of hypercalcemia of malignancy: A pooled analysis of two randomized, controlled clinical trials. J. Clin. Oncol..

[49] Hu M.I., Glezerman I.G., Leboulleux S., Insogna K., Gucalp R., Misiorowski W., Yu B., Zorsky P., Tosi D., Bessudo A. (2014). Denosumab for treatment of hypercalcemia of malignancy. J. Clin. Endocrinol. Metab..

[50] Stopeck A.T., Fizazi K., Body J.J., Brown J.E., Carducci M., Diel I., Fujiwara Y., Martin M., Paterson A., Tonkin K. (2016). Safety of long-term denosumab therapy: Results from the open label extension phase of two phase 3 studies in patients with metastatic breast and prostate cancer. Support. Care Cancer.

[51] Himelstein A.L., Foster J.C., Khatcheressian J.L., Roberts J.D., Seisler D.K., Novotny P.J., Qin R., Go R.S., Grubbs S.S., O’Connor T. (2017). Effect of Longer-Interval vs Standard Dosing of Zoledronic Acid on Skeletal Events in Patients with Bone Metastases: A Randomized Clinical Trial. JAMA.

[52] Raje N., Vescio R., Montgomery C.W., Badros A., Munshi N., Orlowski R., Hadala J.T., Warsi G., Argonza-Aviles E., Ericson S.G. (2016). Bone Marker-Directed Dosing of Zoledronic Acid for the Prevention of Skeletal Complications in Patients with Multiple Myeloma: Results of the Z-MARK Study. Clin. Cancer Res..

[53] Patel C.G., Yee A.J., Scullen T.A., Nemani N., Santo L., Richardson P.G., Laubach J.P., Ghobrial I.M., Schlossman R.L., Munshi N.C. (2014). Biomarkers of bone remodeling in multiple myeloma patients to tailor bisphosphonate therapy. Clin. Cancer Res..

[54] Abousaud A.I., Barbee M.S., Davis C.C., Caulfield S.E., Wang Z., Boykin A., Carthon B.C., Gogineni K. (2020). Safety and efficacy of extended dosing intervals of denosumab in patients with solid cancers and bone metastases: A retrospective study. Ther. Adv. Med. Oncol..

[55] Badros A., Terpos E., Katodritou E., Goloubeva O., Kastritis E., Verrou E., Zervas K., Baer M.R., Meiller T., Dimopoulos M.A. (2008). Natural history of osteonecrosis of the jaw in patients with multiple myeloma. J. Clin. Oncol..

[56] Fulciniti M., Tassone P., Hideshima T., Vallet S., Nanjappa P., Ettenberg S.A., Shen Z., Patel N., Tai Y.T., Chauhan D. (2009). Anti-DKK1 mAb (BHQ880) as a potential therapeutic agent for multiple myeloma. Blood.

[57] Pozzi S., Fulciniti M., Yan H., Vallet S., Eda H., Patel K., Santo L., Cirstea D., Hideshima T., Schirtzinge L. (2013). In vivo and in vitro effects of a novel anti-Dkk1 neutralizing antibody in multiple myeloma. Bone.

[58] Vallet S., Mukherjee S., Vaghela N., Hideshima T., Fulciniti M., Pozzi S., Santo L., Cirstea D., Patel K., Sohani A.R. (2010). Activin A promotes multiple myeloma-induced osteolysis and is a promising target for myeloma bone disease. Proc. Natl. Acad. Sci. USA.

[59] McDonald M.M., Reagan M.R., Youlten S.E., Mohanty S.T., Seckinger A., Terry R.L., Pettitt J.A., Simic M.K., Cheng T.L., Morse A. (2017). Inhibiting the osteocyte-specific protein sclerostin increases bone mass and fracture resistance in multiple myeloma. Blood.

[60] Iyer S.P., Beck J.T., Stewart A.K., Shah J., Kelly K.R., Isaacs R., Bilic S., Sen S., Munshi N.C. (2014). A Phase IB multicentre dose-determination study of BHQ880 in combination with anti-myeloma therapy and zoledronic acid in patients with relapsed or refractory multiple myeloma and prior skeletal-related events. Br. J. Haematol..

[61] Blimark C., Holmberg E., Mellqvist U.H., Landgren O., Bjorkholm M., Hultcrantz M., Kjellander C., Turesson I., Kristinsson S.Y. (2015). Multiple myeloma and infections: A population-based study on 9253 multiple myeloma patients. Haematologica.

[62] Pratt G., Goodyear O., Moss P. (2007). Immunodeficiency and immunotherapy in multiple myeloma. Br. J. Haematol..

[63] Chakraborty R., Rybicki L., Nakashima M.O., Dean R.M., Faiman B.M., Samaras C.J., Rosko N., Dysert H., Valent J., Anwer F. (2020). Characterisation and prognostic impact of immunoparesis in relapsed multiple myeloma. Br. J. Haematol..

[64] Teh B.W., Harrison S.J., Pellegrini M., Thursky K.A., Worth L.J., Slavin M.A. (2014). Changing treatment paradigms for patients with plasma cell myeloma: Impact upon immune determinants of infection. Blood Rev..

[65] Nahi H., Chrobok M., Gran C., Lund J., Gruber A., Gahrton G., Ljungman P., Wagner A.K., Alici E. (2019). Infectious complications and NK cell depletion following daratumumab treatment of Multiple Myeloma. PLoS ONE.

[66] Basler M., Lauer C., Beck U., Groettrup M. (2009). The proteasome inhibitor bortezomib enhances the susceptibility to viral infection. J. Immunol..

[67] Lim C., Sinha P., Harrison S.J., Quach H., Slavin M.A., Teh B.W. (2021). Epidemiology and Risks of Infections in Patients With Multiple Myeloma Managed With New Generation Therapies. Clin. Lymphoma Myeloma Leuk..

[68] Ludwig H., Boccadoro M., Moreau P., San-Miguel J., Cavo M., Pawlyn C., Zweegman S., Facon T., Driessen C., Hajek R. (2021). Recommendations for vaccination in multiple myeloma: A consensus of the European Myeloma Network. Leukemia.

[69] Dimopoulos M.A., Moreau P., Terpos E., Mateos M.V., Zweegman S., Cook G., Delforge M., Hajek R., Schjesvold F., Cavo M. (2021). Multiple Myeloma: EHA-ESMO Clinical Practice Guidelines for Diagnosis, Treatment and Follow-Up. Hemasphere.

[70] Vickrey E., Allen S., Mehta J., Singhal S. (2009). Acyclovir to prevent reactivation of varicella zoster virus (herpes zoster) in multiple myeloma patients receiving bortezomib therapy. Cancer.

[71] Cunningham A.L., Lal H., Kovac M., Chlibek R., Hwang S.J., Diez-Domingo J., Godeaux O., Levin M.J., McElhaney J.E., Puig-Barbera J. (2016). Efficacy of the Herpes Zoster Subunit Vaccine in Adults 70 Years of Age or Older. N. Engl. J. Med..

[72] Dagnew A.F., Ilhan O., Lee W.S., Woszczyk D., Kwak J.Y., Bowcock S., Sohn S.K., Rodriguez Macias G., Chiou T.J., Quiel D. (2019). Immunogenicity and safety of the adjuvanted recombinant zoster vaccine in adults with haematological malignancies: A phase 3, randomised, clinical trial and post-hoc efficacy analysis. Lancet Infect. Dis..

[73] Sweiss K., Calip G., Galvin J., Rondelli D., Patel P. (2019). High Rates of Varicella Zoster Virus Antibody Seroconversion after Administration of the Adjuvanted, Recombinant Varicella Zoster Vaccine in Multiple Myeloma Patients Undergoing Active Treatment. Blood.

[74] Raanani P., Gafter-Gvili A., Paul M., Ben-Bassat I., Leibovici L., Shpilberg O. (2009). Immunoglobulin prophylaxis in chronic lymphocytic leukemia and multiple myeloma: Systematic review and meta-analysis. Leuk. Lymphoma.

[75] Bonilla F.A. (2016). Intravenous and subcutaneous immunoglobulin G replacement therapy. Allergy Asthma Proc..

[76] Vacca A., Melaccio A., Sportelli A., Solimando A.G., Dammacco F., Ria R. (2018). Subcutaneous immunoglobulins in patients with multiple myeloma and secondary hypogammaglobulinemia: A randomized trial. Clin. Immunol..

[77] Brown J.E., Wood S.L., Confavreux C., Abe M., Weilbaecher K., Hadji P., Johnson R.W., Rhoades J.A., Edwards C.M., Croucher P.I. (2021). Management of bone metastasis and cancer treatment-induced bone loss during the COVID-19 pandemic: An international perspective and recommendations. J. Bone Oncol..

[78] Martinez-Lopez J., Mateos M.V., Encinas C., Sureda A., Hernandez-Rivas J.A., Lopez de la Guia A., Conde D., Krsnik I., Prieto E., Riaza Grau R. (2020). Multiple myeloma and SARS-CoV-2 infection: Clinical characteristics and prognostic factors of inpatient mortality. Blood Cancer J..

[79] Chari A., Samur M.K., Martinez-Lopez J., Cook G., Biran N., Yong K., Hungria V., Engelhardt M., Gay F., Garcia Feria A. (2020). Clinical features associated with COVID-19 outcome in multiple myeloma: First results from the International Myeloma Society data set. Blood.

[80] (2021). Recommendations for Anti-Covid-19 Vaccination in Patients with Multiple Myeloma (MM) and Related Conditions, AL Amyloidosis and Other Monoclonal Gammopathies of Clinical Significance. https://myelomasociety.org/wp-content/uploads/2021/03/PM-COVID-vaccination-in-MM-guidelines-The-Final.pdf.

[81] Ludwig H., San-Miguel J., Munshi N., Sonneveld P., Mateos M.V., Moreau P., Terpos E. (2021). Covid-19 vaccination in patients with multiple myeloma: Focus on immune response. Am. J. Hematol..

[82] Terpos E., Trougakos I.P., Gavriatopoulou M., Papassotiriou I., Sklirou A.D., Ntanasis-Stathopoulos I., Papanagnou E.D., Fotiou D., Kastritis E., Dimopoulos M.A. (2021). Low Neutralizing Antibody Responses Against SARS-CoV-2 in Elderly Myeloma Patients After the First BNT162b2 Vaccine Dose. Blood.

[83] Pimpinelli F., Marchesi F., Piaggio G., Giannarelli D., Papa E., Falcucci P., Pontone M., Di Martino S., Laquintana V., La Malfa A. (2021). Fifth-week immunogenicity and safety of anti-SARS-CoV-2 BNT162b2 vaccine in patients with multiple myeloma and myeloproliferative malignancies on active treatment: Preliminary data from a single institution. J. Hematol. Oncol..

[84] Weber D.M., Chen C., Niesvizky R., Wang M., Belch A., Stadtmauer E.A., Siegel D., Borrello I., Rajkumar S.V., Chanan-Khan A.A. (2007). Lenalidomide plus dexamethasone for relapsed multiple myeloma in North America. N. Engl. J. Med..

[85] Miguel J.S., Weisel K., Moreau P., Lacy M., Song K., Delforge M., Karlin L., Goldschmidt H., Banos A., Oriol A. (2013). Pomalidomide plus low-dose dexamethasone versus high-dose dexamethasone alone for patients with relapsed and refractory multiple myeloma (MM-003): A randomised, open-label, phase 3 trial. Lancet Oncol..

[86] Richardson P.G., Sonneveld P., Schuster M.W., Irwin D., Stadtmauer E.A., Facon T., Harousseau J.L., Ben-Yehuda D., Lonial S., Goldschmidt H. (2005). Bortezomib or high-dose dexamethasone for relapsed multiple myeloma. N. Engl. J. Med..

[87] Moreau P., Masszi T., Grzasko N., Bahlis N.J., Hansson M., Pour L., Sandhu I., Ganly P., Baker B.W., Jackson S.R. (2016). Oral Ixazomib, Lenalidomide, and Dexamethasone for Multiple Myeloma. N. Engl. J. Med..

[88] Lonial S., Dimopoulos M., Palumbo A., White D., Grosicki S., Spicka I., Walter-Croneck A., Moreau P., Mateos M.V., Magen H. (2015). Elotuzumab Therapy for Relapsed or Refractory Multiple Myeloma. N. Engl. J. Med..

[89] Dimopoulos M.A., Oriol A., Nahi H., San-Miguel J., Bahlis N.J., Usmani S.Z., Rabin N., Orlowski R.Z., Komarnicki M., Suzuki K. (2016). Daratumumab, Lenalidomide, and Dexamethasone for Multiple Myeloma. N. Engl. J. Med..

[90] Mikhael J., Richter J., Vij R., Cole C., Zonder J., Kaufman J.L., Bensinger W., Dimopoulos M., Lendvai N., Hari P. (2020). A dose-finding Phase 2 study of single agent isatuximab (anti-CD38 mAb) in relapsed/refractory multiple myeloma. Leukemia.

[91] Lonial S., Lee H.C., Badros A., Trudel S., Nooka A.K., Chari A., Abdallah A.O., Callander N., Lendvai N., Sborov D. (2020). Belantamab mafodotin for relapsed or refractory multiple myeloma (DREAMM-2): A two-arm, randomised, open-label, phase 2 study. Lancet Oncol..

[92] San-Miguel J.F., Hungria V.T., Yoon S.S., Beksac M., Dimopoulos M.A., Elghandour A., Jedrzejczak W.W., Gunther A., Nakorn T.N., Siritanaratkul N. (2014). Panobinostat plus bortezomib and dexamethasone versus placebo plus bortezomib and dexamethasone in patients with relapsed or relapsed and refractory multiple myeloma: A multicentre, randomised, double-blind phase 3 trial. Lancet Oncol..

[93] Richardson P.G., Delforge M., Beksac M., Wen P., Jongen J.L., Sezer O., Terpos E., Munshi N., Palumbo A., Rajkumar S.V. (2012). Management of treatment-emergent peripheral neuropathy in multiple myeloma. Leukemia.

[94] Richardson P.G., Briemberg H., Jagannath S., Wen P.Y., Barlogie B., Berenson J., Singhal S., Siegel D.S., Irwin D., Schuster M. (2006). Frequency, characteristics, and reversibility of peripheral neuropathy during treatment of advanced multiple myeloma with bortezomib. J. Clin. Oncol..

[95] Yamamoto S., Egashira N. (2021). Pathological Mechanisms of Bortezomib-Induced Peripheral Neuropathy. Int. J. Mol. Sci..

[96] Carozzi V.A., Renn C.L., Bardini M., Fazio G., Chiorazzi A., Meregalli C., Oggioni N., Shanks K., Quartu M., Serra M.P. (2013). Bortezomib-induced painful peripheral neuropathy: An electrophysiological, behavioral, morphological and mechanistic study in the mouse. PLoS ONE.

[97] Gendreau S., Berzero G., Tafani C., Raynouard I., Ricard D., Malfuson J.V., Viala K., Debs R., Houillier C., Diamanti L. (2020). Demyelinating polyradiculoneuritis in patients with multiple myeloma: The other side of bortezomib-induced neurotoxicity. Acta Oncol..

[98] Thawani S.P., Tanji K., De Sousa E.A., Weimer L.H., Brannagan T.H. (2015). Bortezomib-associated demyelinating neuropathy—Clinical and pathologic features. J. Clin. Neuromuscul. Dis..

[99] Ohi T., Kyle R.A., Dyck P.J. (1985). Axonal attenuation and secondary segmental demyelination in myeloma neuropathies. Ann. Neurol..

[100] Denier C., Lozeron P., Adams D., Decaudin D., Isnard-Grivaux F., Lacroix C., Said G. (2006). Multifocal neuropathy due to plasma cell infiltration of peripheral nerves in multiple myeloma. Neurology.

[101] Minarik J., Pavlicek P., Pour L., Pika T., Maisnar V., Spicka I., Jarkovsky J., Krejci M., Bacovsky J., Radocha J. (2015). Subcutaneous bortezomib in multiple myeloma patients induces similar therapeutic response rates as intravenous application but it does not reduce the incidence of peripheral neuropathy. PLoS ONE.

[102] Corso A., Mangiacavalli S., Varettoni M., Pascutto C., Zappasodi P., Lazzarino M. (2010). Bortezomib-induced peripheral neuropathy in multiple myeloma: A comparison between previously treated and untreated patients. Leuk. Res..

[103] Richardson P.G., Sonneveld P., Schuster M.W., Stadtmauer E.A., Facon T., Harousseau J.L., Ben-Yehuda D., Lonial S., Goldschmidt H., Reece D. (2009). Reversibility of symptomatic peripheral neuropathy with bortezomib in the phase III APEX trial in relapsed multiple myeloma: Impact of a dose-modification guideline. Br. J. Haematol..

[104] Finnerup N.B., Attal N., Haroutounian S., McNicol E., Baron R., Dworkin R.H., Gilron I., Haanpaa M., Hansson P., Jensen T.S. (2015). Pharmacotherapy for neuropathic pain in adults: A systematic review and meta-analysis. Lancet Neurol..

[105] Zhi W.I., Ingram E., Li S.Q., Chen P., Piulson L., Bao T. (2018). Acupuncture for Bortezomib-Induced Peripheral Neuropathy: Not Just for Pain. Integr. Cancer Ther..

[106] Kristinsson S.Y., Fears T.R., Gridley G., Turesson I., Mellqvist U.H., Bjorkholm M., Landgren O. (2008). Deep vein thrombosis after monoclonal gammopathy of undetermined significance and multiple myeloma. Blood.

[107] Fotiou D., Sergentanis T.N., Papageorgiou L., Stamatelopoulos K., Gavriatopoulou M., Kastritis E., Psaltopoulou T., Salta S., Van Dreden P., Sangare R. (2018). Longer procoagulant phospholipid-dependent clotting time, lower endogenous thrombin potential and higher tissue factor pathway inhibitor concentrations are associated with increased VTE occurrence in patients with newly diagnosed multiple myeloma: Results of the prospective ROADMAP-MM-CAT study. Blood Cancer J..

[108] Dimopoulos M.A., Chen C., Spencer A., Niesvizky R., Attal M., Stadtmauer E.A., Petrucci M.T., Yu Z., Olesnyckyj M., Zeldis J.B. (2009). Long-term follow-up on overall survival from the MM-009 and MM-010 phase III trials of lenalidomide plus dexamethasone in patients with relapsed or refractory multiple myeloma. Leukemia.

[109] Anagnostopoulos A., Weber D., Rankin K., Delasalle K., Alexanian R. (2003). Thalidomide and dexamethasone for resistant multiple myeloma. Br. J. Haematol..

[110] Lee C.K., Barlogie B., Munshi N., Zangari M., Fassas A., Jacobson J., van Rhee F., Cottler-Fox M., Muwalla F., Tricot G. (2003). DTPACE: An effective, novel combination chemotherapy with thalidomide for previously treated patients with myeloma. J. Clin. Oncol..

[111] Shin J., Lee J.J., Kim K., Min C.K., Lee J.O., Suh C., Kim J.S., Lee Y.J., Yoon S.S., Jo J.C. (2019). Venous thromboembolism in relapsed or refractory multiple myeloma patients treated with lenalidomide plus dexamethasone. Int. J. Hematol..

[112] Murakami H., Shimizu K., Sawamura M., Suzuki K., Sugiura I., Kosugi H., Shimazaki C., Taniwaki M., Abe M., Takagi T. (2009). Phase II and pharmacokinetic study of thalidomide in Japanese patients with relapsed/refractory multiple myeloma. Int. J. Hematol..

[113] Palumbo A., Rajkumar S.V., Dimopoulos M.A., Richardson P.G., San Miguel J., Barlogie B., Harousseau J., Zonder J.A., Cavo M., Zangari M. (2008). Prevention of thalidomide- and lenalidomide-associated thrombosis in myeloma. Leukemia.

[114] McCarthy P.L., Owzar K., Hofmeister C.C., Hurd D.D., Hassoun H., Richardson P.G., Giralt S., Stadtmauer E.A., Weisdorf D.J., Vij R. (2012). Lenalidomide after stem-cell transplantation for multiple myeloma. N. Engl. J. Med..

[115] Key N.S., Khorana A.A., Kuderer N.M., Bohlke K., Lee A.Y.Y., Arcelus J.I., Wong S.L., Balaban E.P., Flowers C.R., Francis C.W. (2020). Venous Thromboembolism Prophylaxis and Treatment in Patients with Cancer: ASCO Clinical Practice Guideline Update. J. Clin. Oncol..

[116] Sanfilippo K.M., Luo S., Wang T.F., Fiala M., Schoen M., Wildes T.M., Mikhael J., Kuderer N.M., Calverley D.C., Keller J. (2019). Predicting venous thromboembolism in multiple myeloma: Development and validation of the IMPEDE VTE score. Am. J. Hematol..

[117] Calafiore V., Giamporcaro S., Conticello C., Romano A., Parisi M., Giuffrida G., Tibullo D., Di Raimondo F., Signorelli S.S. (2020). A Real-Life Survey of Venous Thromboembolic Events Occurring in Myeloma Patients Treated in Third Line with Second-Generation Novel Agents. J. Clin. Med..

[118] Dimopoulos M.A., Swern A.S., Li J.S., Hussein M., Weiss L., Nagarwala Y., Baz R. (2014). Efficacy and safety of long-term treatment with lenalidomide and dexamethasone in patients with relapsed/refractory multiple myeloma. Blood Cancer J..

[119] Richardson P.G., Weller E., Jagannath S., Avigan D.E., Alsina M., Schlossman R.L., Mazumder A., Munshi N.C., Ghobrial I.M., Doss D. (2009). Multicenter, phase I, dose-escalation trial of lenalidomide plus bortezomib for relapsed and relapsed/refractory multiple myeloma. J. Clin. Oncol..

[120] Chakraborty R., Bin Riaz I., Malik S.U., Marneni N., Garcia A.M., Anwer F., Khorana A.A., Rajkumar S.V., Kumar S., Murad M.H. (2020). Venous thromboembolism risk with contemporary lenalidomide-based regimens despite thromboprophylaxis in multiple myeloma: A systematic review and meta-analysis. Cancer.

[121] Cornell R.F., Goldhaber S.Z., Engelhardt B.G., Moslehi J., Jagasia M., Harrell S., Rubinstein S.M., Hall R., Wyatt H., Piazza G. (2020). Primary prevention of venous thromboembolism with apixaban for multiple myeloma patients receiving immunomodulatory agents. Br. J. Haematol..

[122] Pegourie B., Karlin L., Benboubker L., Orsini-Piocelle F., Tiab M., Auger-Quittet S., Rodon P., Royer B., Leleu X., Bareau B. (2019). Apixaban for the prevention of thromboembolism in immunomodulatory-treated myeloma patients: Myelaxat, a phase 2 pilot study. Am. J. Hematol..

[123] Fakhri B., Fiala M.A., Shah N., Vij R., Wildes T.M. (2020). Measuring cardiopulmonary complications of carfilzomib treatment and associated risk factors using the SEER-Medicare database. Cancer.

[124] Ling Y., Li R., Zhong J., Zhao Y., Chen Z. (2020). Ixazomib-associated cardiovascular adverse events in multiple myeloma: A systematic review and meta-analysis. Drug Chem. Toxicol..

[125] Chari A., Stewart A.K., Russell S.D., Moreau P., Herrmann J., Banchs J., Hajek R., Groarke J., Lyon A.R., Batty G.N. (2018). Analysis of carfilzomib cardiovascular safety profile across relapsed and/or refractory multiple myeloma clinical trials. Blood Adv..

[126] Chavda S.J., Pocock R., Cheesman S., Lee K.M., Dowling E., Marks D.J.B., Kyriakou C., Lee L., Sive J., Wechalekar A. (2020). Association of hypertension and cardiac events in patients with multiple myeloma receiving carfilzomib: Practical management recommendations. Br. J. Haematol..

[127] Waxman A.J., Clasen S., Hwang W.T., Garfall A., Vogl D.T., Carver J., O’Quinn R., Cohen A.D., Stadtmauer E.A., Ky B. (2018). Carfilzomib-Associated Cardiovascular Adverse Events: A Systematic Review and Meta-analysis. JAMA Oncol..

[128] Latif A., Kapoor V., Lateef N., Ahsan M.J., Usman R.M., Malik S.U., Ahmad N., Rosko N., Rudoni J., William P. (2021). Incidence and Management of Carfilzomib-induced Cardiovascular Toxicity; A Systematic Review and Meta-analysis. Cardiovasc. Hematol. Disord. Drug Targets.

[129] Bringhen S., Milan A., D’Agostino M., Ferri C., Wasch R., Gay F., Larocca A., Offidani M., Zweegman S., Terpos E. (2019). Prevention, monitoring and treatment of cardiovascular adverse events in myeloma patients receiving carfilzomib A consensus paper by the European Myeloma Network and the Italian Society of Arterial Hypertension. J. Intern. Med..

[130] Hesketh P.J., Kris M.G., Basch E., Bohlke K., Barbour S.Y., Clark-Snow R.A., Danso M.A., Dennis K., Dupuis L.L., Dusetzina S.B. (2020). Antiemetics: ASCO Guideline Update. J. Clin. Oncol..

[131] Pawlyn C., Khan M.S., Muls A., Sriskandarajah P., Kaiser M.F., Davies F.E., Morgan G.J., Andreyev H.J. (2014). Lenalidomide-induced diarrhea in patients with myeloma is caused by bile acid malabsorption that responds to treatment. Blood.

[132] Watson M., Nooka A., Gleason C., Valla K., Kaufman J., Lonial S. (2014). Colesevelam Hydrochloride for the Treatment of Lenalidomide Induced Diarrhea. Blood.

[133] Gavriatopoulou M., Chari A., Chen C., Bahlis N., Vogl D.T., Jakubowiak A., Dingli D., Cornell R.F., Hofmeister C.C., Siegel D. (2020). Integrated safety profile of selinexor in multiple myeloma: Experience from 437 patients enrolled in clinical trials. Leukemia.

[134] Chen C., Siegel D., Gutierrez M., Jacoby M., Hofmeister C.C., Gabrail N., Baz R., Mau-Sorensen M., Berdeja J.G., Savona M. (2018). Safety and efficacy of selinexor in relapsed or refractory multiple myeloma and Waldenstrom macroglobulinemia. Blood.

[135] Desikan K.R., Dhodapkar M.V., Hough A., Waldron T., Jagannath S., Siegel D., Barlogie B., Tricot G. (1997). Incidence and impact of light chain associated (AL) amyloidosis on the prognosis of patients with multiple myeloma treated with autologous transplantation. Leuk. Lymphoma.

[136] Mendelson L., Shelton A., Brauneis D., Sanchorawala V. (2020). AL Amyloidosis in Myeloma: Red Flag Symptoms. Clin. Lymphoma Myeloma Leuk..

[137] Merlini G., Dispenzieri A., Sanchorawala V., Schonland S.O., Palladini G., Hawkins P.N., Gertz M.A. (2018). Systemic immunoglobulin light chain amyloidosis. Nat. Rev. Dis. Primers.

[138] Seo M., Cha H.J., Kim M., Park S.H., Lim J.H., Choi Y., Lee Y.J., Park S.H., Jo J.C. (2019). Clinical Utility of 18F-Florbetaben PET for Detecting Amyloidosis Associated With Multiple Myeloma: A Prospective Case-Control Study. Clin. Nucl. Med..

[139] Sklar B.A., Gervasio K.A., Leng S., Ghosh A., Chari A., Wu A.Y. (2019). Management and outcomes of proteasome inhibitor associated chalazia and blepharitis: A case series. BMC Ophthalmol..

[140] Pennisi M., Berchicci L., Miserocchi E., Mussetti A., Cacioppo V., David A., Scialdone A., Lorusso I., Modorati G., Corradini P. (2019). Ocular disorders in multiple myeloma patients: Cross-sectional study of prevalence and association with treatment. Leuk. Lymphoma.

[141] Bausell R.B., Soleimani A., Vinnett A., Baroni M.D., Staub S.A., Binion K., Jeng B.H., Badros A.Z., Munir W.M. (2021). Corneal Changes after Belantamab Mafodotin in Multiple Myeloma Patients. Eye Contact Lens.

[142] Farooq A.V., Degli Esposti S., Popat R., Thulasi P., Lonial S., Nooka A.K., Jakubowiak A., Sborov D., Zaugg B.E., Badros A.Z. (2020). Corneal Epithelial Findings in Patients with Multiple Myeloma Treated with Antibody-Drug Conjugate Belantamab Mafodotin in the Pivotal, Randomized, DREAMM-2 Study. Ophthalmol. Ther..

[143] Lonial S., Nooka A.K., Thulasi P., Badros A.Z., Jeng B.H., Callander N.S., Potter H.A., Sborov D., Zaugg B.E., Popat R. (2021). Management of belantamab mafodotin-associated corneal events in patients with relapsed or refractory multiple myeloma (RRMM). Blood Cancer J..

[144] Giri S., Zhu W., Wang R., Zeidan A., Podoltsev N., Gore S.D., Neparidze N., Ma X., Gross C.P., Davidoff A.J. (2019). Underutilization of guideline-recommended supportive care among older adults with multiple myeloma in the United States. Cancer.

[145] Tournaire G., Conte C., Perrot A., Lapeyre-Mester M., Despas F. (2020). Vaccination during the First Diagnosis of Multiple Myeloma: A Cohort Study of the French National Health Insurance Database. Vaccines.

[146] Chari A., Romanus D., Palumbo A., Blazer M., Farrelly E., Raju A., Huang H., Richardson P. (2020). Randomized Clinical Trial Representativeness and Outcomes in Real-World Patients: Comparison of 6 Hallmark Randomized Clinical Trials of Relapsed/Refractory Multiple Myeloma. Clin. Lymphoma Myeloma Leuk..

[147] Usami E., Kimura M., Takenaka S., Iwai M., Teramachi H., Yoshimura T. (2019). Tolerability and safety of real-world use of pomalidomide in patients with relapsed/refractory multiple myeloma. Mol. Clin. Oncol..

[148] Beaver J.A., Ison G., Pazdur R. (2017). Reevaluating Eligibility Criteria—Balancing Patient Protection and Participation in Oncology Trials. N. Engl. J. Med..

[149] Raje N., Berdeja J., Lin Y., Siegel D., Jagannath S., Madduri D., Liedtke M., Rosenblatt J., Maus M.V., Turka A. (2019). Anti-BCMA CAR T-Cell Therapy bb2121 in Relapsed or Refractory Multiple Myeloma. N. Engl. J. Med..

[150] Munshi N.C., Anderson L.D., Shah N., Madduri D., Berdeja J., Lonial S., Raje N., Lin Y., Siegel D., Oriol A. (2021). Idecabtagene Vicleucel in Relapsed and Refractory Multiple Myeloma. N. Engl. J. Med..

[151] Topp M.S., Duell J., Zugmaier G., Attal M., Moreau P., Langer C., Kronke J., Facon T., Salnikov A.V., Lesley R. (2020). Anti-B-Cell Maturation Antigen BiTE Molecule AMG 420 Induces Responses in Multiple Myeloma. J. Clin. Oncol..

[152] Swan D., Routledge D., Harrison S. (2021). The evolving status of immunotherapies in multiple myeloma: The future role of bispecific antibodies. Br. J. Haematol..

[153] Van de Donk N., Usmani S.Z., Yong K. (2021). CAR T-cell therapy for multiple myeloma: State of the art and prospects. Lancet Haematol..

[154] Usmani S.Z., Garfall A.L., van de Donk N., Nahi H., San-Miguel J.F., Oriol A., Rosinol L., Chari A., Bhutani M., Karlin L. (2021). Teclistamab, a B-cell maturation antigen × CD3 bispecific antibody, in patients with relapsed or refractory multiple myeloma (MajesTEC-1): A multicentre, open-label, single-arm, phase 1 study. Lancet.

[155] Cohen A.D., Garfall A.L., Stadtmauer E.A., Melenhorst J.J., Lacey S.F., Lancaster E., Vogl D.T., Weiss B.M., Dengel K., Nelson A. (2019). B cell maturation antigen-specific CAR T cells are clinically active in multiple myeloma. J. Clin. Investig..

[156] Neelapu S.S. (2019). Managing the toxicities of CAR T-cell therapy. Hematol. Oncol..

[157] Gonzalez-Rodriguez E., Aubry-Rozier B., Stoll D., Zaman K., Lamy O. (2020). Sixty spontaneous vertebral fractures after denosumab discontinuation in 15 women with early-stage breast cancer under aromatase inhibitors. Breast Cancer Res. Treat..

[158] Kotchetkov R., Masih-Khan E., Chu C.M., Atenafu E.G., Chen C., Kukreti V., Trudel S., Tiedemann R., Reece D.E. (2017). Secondary primary malignancies during the lenalidomide-dexamethasone regimen in relapsed/refractory multiple myeloma patients. Cancer Med..

